# Effect of Yttria Content on the Translucency and Masking Ability of Yttria-Stabilized Tetragonal Zirconia Polycrystal

**DOI:** 10.3390/ma13214726

**Published:** 2020-10-22

**Authors:** Young-Eun Cho, Young-Jun Lim, Jung-Suk Han, In-Sung Luke Yeo, Hyung-In Yoon

**Affiliations:** 1 Department of Prosthodontics, Dankook University Dental Hospital, Cheonan 31116, Korea; yecho77@hanmail.net; 2 Department of Prosthodontics and Dental Research Institute, School of Dentistry, Seoul National University, Seoul 03080, Korea; pros53@snu.ac.kr (I.-S.L.Y.); drhiy226@snu.ac.kr (H.-I.Y.)

**Keywords:** color difference, masking ability, translucency parameters, visual perception, yttria-stabilized zirconia

## Abstract

Translucent zirconia, manufactured by increasing the yttria content, offers improved translucency, but may have a negative effect on esthetic outcomes under clinical conditions such as discolored abutment because of the reflection of the underlying color. The purpose of this in vitro study was to compare the translucency parameter and masking ability of 3 mol % yttria-stabilized tetragonal zirconia polycrystal (3Y-TZP (Katana HT)), 4Y-ZP (Katana STML), and 5Y-ZP (Katana UTML) with those of lithium disilicate (Rosetta SM). Zirconia and lithium disilicate specimens of 10 mm diameters and 0.8 and 1.5 mm thicknesses were fabricated. Their CIE L*a*b* values (L*, brightness; a*, red-green value; b*, yellow-blue value) were measured at the center of the specimens against black and white backgrounds using a spectrophotometer, and translucency parameter (TP) values were determined. The microstructure of the specimens was observed using scanning electron microscopy. Four cylindrical backgrounds of different shades were prepared. The zirconia and lithium disilicate specimens were placed on the backgrounds without any intervening medium. CIE L*a*b* values were obtained, and the color difference value (ΔE) was calculated. Thresholds for acceptability and perceptibility were assumed as ΔE = 5.5 and ΔE = 2.6, respectively, to evaluate masking ability. Data were compared using one-way analysis of variance and post-hoc was performed using Scheffe’s test (α = 0.05). In zirconia specimens, the TP value increased as the yttria content increased from 3 mol %, through 4 mol % to 5 mol %, and all zirconia specimens showed lower TP values than lithium disilicate specimens did. All zirconia specimens showed optimal masking ability against a normal dentin shade (ND3) and acceptable masking ability against titanium at a minimum thickness of 1.5 mm. However, no zirconia specimen could mask severely discolored dentin (ND9), regardless of thickness. The decrease in zirconia thickness from 1.5 to 0.8 mm significantly increased translucency. Monolithic Y-TZP ceramics could mask a normal dentin background but could not mask severely discolored dentin at either 0.8 or 1.5 mm thicknesses.

## 1. Introduction

Zirconia is a bioinert and biocompatible material with high mechanical strength, good chemical stability, and low thermal conductivity [1,2,3,4]. For stable use of zirconia in dentistry, 3 mol % yttria is added to stabilize the tetragonal phase of zirconia at room temperature [5,6]. Three mole percent yttria-stabilized tetragonal zirconia polycrystal (3Y-TZP) responds to crack formation by transformation toughening, which results in high fracture toughness [5].

Despite its excellent mechanical properties, the opaque nature of 3Y-TZP is its significant disadvantage. This esthetic deficiency has conventionally been managed by the use of a layer of powder-fire porcelain on a zirconia core, but bilayered structures are vulnerable to chipping and delamination, exacerbated by thermally induced residual stresses [6]. Attempts to minimize this vulnerability have been made by milling the veneer and the framework separately and then joining them with either resin luting agents or fusion firing. However, luting agents are compliant, allowing flexure of the veneer, and fusion firing does not eliminate residual stresses [7]. In addition, veneering increases restoration thickness, which requires a greater amount of tooth preparation. Therefore, considerable attention has been focused on monolithic restorations because of the simplicity in fabrication and reduced material thickness, with efforts to satisfy the durability and esthetic requirements.

In an attempt to develop a monolithic ceramic with acceptable translucency, 3Y-TZP processing was refined by reducing the concentration of alumina and minimizing the porosities by sintering at a higher temperature [7,8]. Using this method, translucency improved to some extent, but restorations were still insufficiently esthetic for use in the anterior zone.

More recently, dental zirconia has been manufactured with increased yttria content to overcome the esthetic deficiencies. Partially stabilized zirconia with higher yttria content comprises an increased nonbirefringent cubic phase [7]. Doping zirconia with up to 5 mol % yttria creates partially stabilized zirconia with approximately 50% cubic phase (5Y-ZP) [5,6]. The cubic phase of zirconia is isotropic in different crystallographic directions, which decreases light scattering at grain boundaries. As a result, cubic zirconia with yttria content higher than that of 3Y-TZP appears more translucent [9,10].

However, translucent materials may have a negative effect on esthetic outcomes under clinical conditions such as discolored tooth abutment, metal core, or titanium implant abutment, as the underlying color would be reflected. The effects of the background on the color of porcelain veneers [11,12] and lithium disilicate ceramics [13,14,15,16] have been investigated, but limited information is available on the effects of specific backgrounds on the color of translucent zirconia.

Therefore, the purpose of this in vitro study was to compare the translucency parameter and masking ability of zirconia materials exhibiting differing yttria contents with those of lithium disilicate ceramics. First, we compared the translucency of 3Y-TZP (Katana HT), 4Y-ZP (Katana STML), and 5Y-ZP (Katama UTML) with that of lithium disilicate (Rosetta SM). Thereafter, we compared the masking ability of these zirconia materials with that of lithium disilicate on three different backgrounds.

## 2. Materials and Methods

### 2.1. Preparation of the Zirconia Specimens

**Figure 1:**
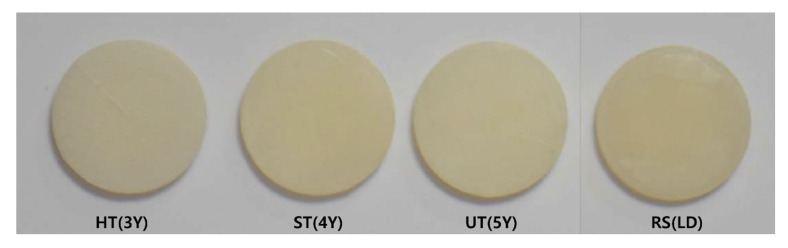
Tested ceramic specimens. HT(3Y), Katana HT (3 mol % Y_2_O_3_ Tetragonal Zirconia Polycrystals); ST(4Y), Katana STML (4 mol % Y_2_O_3_ Zirconia Polycrystals); UT(5Y), Katana UTML (5 mol % Y_2_O_3_ Zirconia Polycrystals); RS(LD), Rosetta SM (lithium disilicate).

The zirconia materials selected for this study were Katana HT, STML, and UTML (Kuraray Noritake Dental Inc., Miyoshi, Japan), exhibiting differing yttria content. Rosetta SM (Hass Corp, Gangeung, Korea) was selected as the reference lithium disilicate material (Figure 1). The shade of all specimens was A2. A total of 80 disk-shaped specimens were fabricated and divided into eight groups according to the product and thickness (Table 1). The specimens (n = 10/group) were prepared by sectioning the respective ceramics into 10 mm diameter disks with 0.8 and 1.5 mm thicknesses, using a circular sectioning blade and silicon-carbide abrasive paper. They were sintered according to the manufacturer’s recommendations and polished to the final thickness of 0.8 or 1.5 mm using 1200-grit silicon-carbide paper. Lithium disilicate specimens were wet-sectioned and zirconia specimens were dry-sectioned to replicate the fabrication procedures performed in a dental laboratory. All dimensions were confirmed to be accurate within 0.01 mm using digital calipers. As Katana STML and Katana UTML are multilayered materials, the occlusal surface of the disk was selected to face the spectrophotometer.

### 2.2. Preparation of the Background Substrates

**Figure 2:**
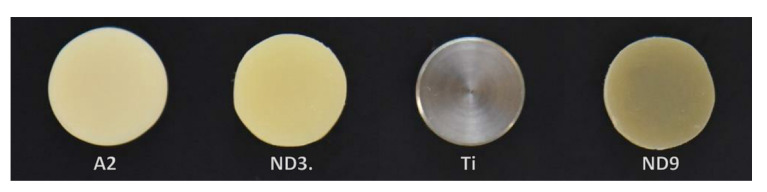
Background substrates for comparing masking ability. A2, A2-shade composite resin (reference); ND3, ND3-shade composite resin (normal dentin); Ti, milled titanium (titanium abutment); ND9, ND9-shade composite resin (severely discolored dentin).

Four backgrounds of different shades were prepared to compare masking ability. Cylindrical substrates simulating different backgrounds included A2-shade composite resin (Filtek Z350, 3M ESPE, St. Paul, MN, USA) for reference, ND3-shade composite resin (IPS natural die material, Ivoclar Vivadent, Schaan, Liechtenstein) for normal dentin, ND9-shade composite resin (IPS natural die material, Ivoclar Vivadent AG, Schaan, Liechtenstein) for severely discolored dentin, and milled titanium for implant abutment (Figure 2). All background substrates were 10 mm in diameter and 5 mm in height. The composite-resin background specimens were prepared by incremental additions of light-curing composite resin in a putty mold, with incremental light-curing for 40 s using a light-polymerizing unit (Elipar Freelight; 3M ESPE, St. Paul, MN, USA). After the composite was cured, it was polished with a silicon-carbide bur.

### 2.3. Evaluation of the Translucency Parameter

To obtain the value of the translucency parameter (TP), the CIE L*a*b* values (L*, brightness; a*, red-green value; b*, yellow-blue value) were measured at the center of the specimens on white and black backgrounds using a spectrophotometer (SpectroShade Micro, MTH Optic Research AG, Niederhasli, Switzerland). The values of each specimen were measured twice and averaged. The TP value was the color difference between values on the black and white backgrounds of the same specimen. The TP value was calculated using the following formula:

TP = [(L*_B_ − L*_W_)^2^ + (a*_B_ − a*_W_)^2^ + (b*_B_ − b*_W_)^2^]^1/2.^ (1)

The microstructures of the zirconia specimens were observed under a scanning electron microscope (SEM; Apreo S, ThermoFisher Scientific, Waltham, MA, USA). For pretreatment, the specimens were degreased, dried, cleaned, and coated with platinum ions.

### 2.4. Evaluation of Masking Ability

**Figure 3:**
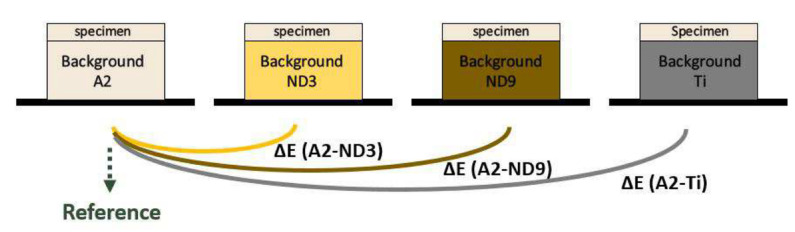
Schematic diagram for evaluation of masking ability. Each specimen was placed on background substrates without intervening medium.

The zirconia and lithium disilicate specimens were placed on four different backgrounds without any intervening medium (Figure 3). The CIE L*a*b* values at the center of the specimens were obtained using a spectrophotometer (SpectroShade Micro). The color difference values (ΔE) were calculated to compare the CIE L*a*b* values against the background A2-shade composite resin. ΔE was calculated using the following formula:

ΔE = [(ΔL*)^2^ + (Δa*)^2^ + (Δb*)^2^]^1/2^ (2)

The thresholds for acceptability and perceptibility were assumed as ΔE = 5.5 and ΔE = 2.6, respectively, to evaluate masking ability [17,18,19].

### 2.5. Statistical Analysis

Statistical software (R Project 3.6.2, R Foundation, Vienna, Austria) was used for statistical analysis of the data. The means of TP and ΔE values for each group were compared using one-way analysis of variance (ANOVA) and post-hoc was performed using Scheffe’s test. The level of statistical significance was set at 0.05.

## 3. Results

### 3.1. Translucency Parameter

For the 0.8 and 1.5 mm thick zirconia specimens, the TP value increased significantly with the increase in yttria content from 3 mol % (HT), through 4 mol % (ST) to 5 mol % (UT). The TP values of zirconia specimens were less than those of lithium disilicate (RS) specimens (Table 2).

For the 0.8 mm thick specimens, the mean TP value of specimens in the HT group (11.58 ± 0.57) was significantly lower than that of those in the ST group (13.90 ± 0.17) (*p* < 0.001), which was significantly lower than the mean TP values in the UT group (15.36 ± 0.50) (*p* < 0.001) and the RS group (19.18 ± 0.29) (*p* < 0.001). There were statistically significant differences between all groups. The transparencies of specimens in the HT, ST, and UT groups were approximately 60%, 73%, and 80%, respectively, that of specimens in the RS group.

As thickness increased, the TP value decreased, implying that opacity increased. In 1.5 mm thick specimens, the mean TP value of specimens in the HT group (7.75 ± 0.57) was significantly lower than that of those in the ST group (11.68 ± 0.23) (*p* < 0.001), the ST group was significantly lower than the UT group (12.64 ± 0.19) (*p* < 0.001), and the UT group was lower than the RS group (14.20 ± 0.39) (*p* < 0.001). There were statistically significant differences between all groups. Similarly, the transparencies of specimens in the HT, ST, and UT groups were approximately 54%, 82%, and 89%, respectively, that of specimens in the RS group.

**Figure 4:**
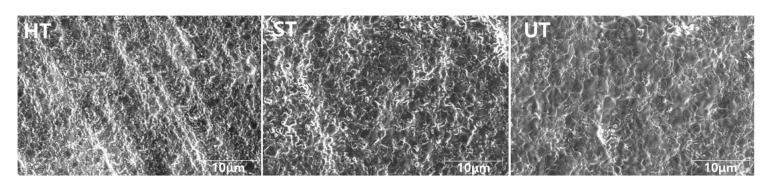
Scanning electron microscope images (original magnification ×10,000) of the tested zirconia specimens. HT, Katana HT; ST, Katana STML; UT, Katana UTML.

Figure 4 shows the representative SEM images of the three groups of zirconia specimens. The SEM image of a representative HT specimen showed a cluster of particles. The microstructures were consistent with yttria and cubic phase content, with HT specimens exhibiting the smallest grains and UT the largest grains, which may have affected the translucency of the specimens.

### 3.2. Masking Ability

**Figure 5:**
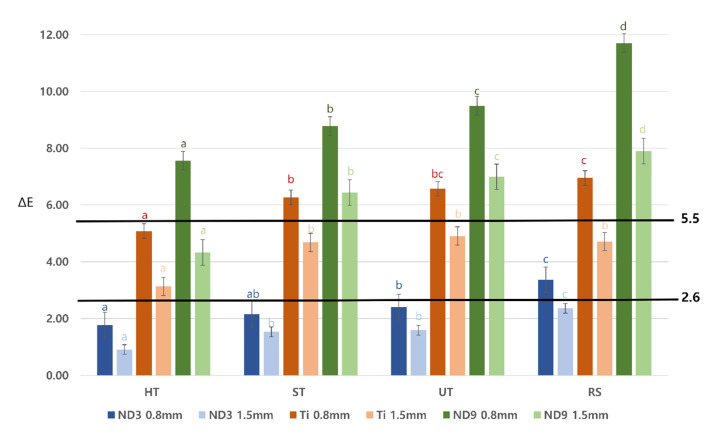
Mean color difference values (ΔE) among the tested materials. Threshold for acceptability ΔE = 5.5, threshold for perceptibility ΔE = 2.6. HT, Katana HT; ST, Katana STML; UT, Katana UTML; RS, Rosetta SM; ND3, ND3-shade composite resin (normal dentin); Ti, milled titanium (titanium abutment); ND9, ND9-shade composite resin (severely discolored dentin); 0.8 and 1.5 mm are the thicknesses of specimens in the respective groups. Different letters (a, b, c, d) on top of the bars mean statistically significant differences between the groups (*p* < 0.05).

Mean ΔE values of the specimens in the zirconia and lithium disilicate groups for the tested backgrounds are presented in Table 3, Table 4 and Table 5 and Figure 5. The results of ANOVA showed that the type of zirconia and its thickness affected ΔE values on different backgrounds (*p* < 0.001). In the zirconia groups, as yttria content increased, ΔE value increased; the RS group showed the highest ΔE value.

Against the ND3 background, ΔE values of the specimens in the zirconia groups were under 2.6, regardless of thickness, implying optimal masking ability (Table 3). The mean ΔE value of 0.8 mm thick specimens in the RS group was 3.36 ± 0.24, which was under 5.5, implying acceptable but not optimal masking ability. On the other hand, in the case of 1.5 mm thick specimens of the RS group, ΔE value was less than 2.6. Therefore, all the tested ceramic materials showed acceptable masking ability against a shade simulating normal dentin.

Against titanium backgrounds, all 1.5 mm thick specimens showed acceptable masking ability (Table 4). However, for the 0.8 mm thick specimens, only those in the HT group (5.08 ± 0.26) showed acceptable masking ability.

Against the ND9 backgrounds, i.e., severely discolored dentin, only 1.5 mm thick specimens of the HT group (4.33 ± 0.45) showed acceptable masking ability (Table 5).

## 4. Discussion

Translucency is defined as the relative amount of light transmission or diffuse reflection from a substrate surface through a turbid medium [20]. The optical appearance of monolithic zirconia dental restorations is influenced by intrinsic (material) and extrinsic (surroundings) parameters. Intrinsic microstructural features, such as grain boundaries and pores, scatter light and result in translucency or opacity [20,21,22].

The translucency and opacity of materials depend on the extent of reflection, scattering, refraction, transmission, and absorption of incident light. Light transmitted to the interior of Y-TZP may exhibit interior reflection and refraction. This phenomenon is termed internal light scattering, which may result from several sources, including pores, impurities, defects, and grain boundaries. [10] In the case of the interaction between light and zirconia particles, some light is reflected when it hits the surface of the zirconia, but most of it is scattered by grain boundaries and internal defects. The rest of the light passes through the pores [23].

The calculation of the translucency parameter is one of the most common approaches to assessing light interactions in dental materials. The translucency parameter has the advantage of direct visual evaluation of translucency [24]. In this study, SpectroShade Micro was used to measure CIE L*a*b* values on white and black backgrounds, and the TP value was calculated. In addition, color difference values (ΔE) on three different backgrounds were calculated to evaluate the masking ability of different mole-percent content Y-TZP materials.

Concerning translucency, thicker ceramic specimens exhibited lower TP values, a finding in line with those of previous studies [25,26]. In addition, the translucency of zirconia specimens (both thicknesses) tended to increase with the increase in yttria content. SEM revealed that specimens of the UT group showed the largest grain size, followed by those of the ST and HT groups (Figure 4). The specimens with larger grains appeared to exhibit more transparency. Therefore, the UT group, which showed larger particles (grains) in SEM analysis, showed higher transparency than the HT group. Since cubic grains are larger than tetragonal grains, these results were consistent with yttria content and cubic fraction. Several studies have also reported the particle sizes of these materials. Putra et al. [27] and Haneada et al. [28] found that the larger the particle size, the more transparent the zirconia, which was also shown by the results of this study.

However, the TP values of the specimens in the Y-TZP groups were lower than those of the specimens in the RS group; hence, monolithic anterior restorations would be limited to the highly translucent lithium disilicate ceramics.

According to Yu et al., the TP value of human enamel at 1 mm thickness is 18.7, and that of dentin is 16.4 [29]. In this study, the TP values of 0.8 mm thick specimens in the HT, ST, and UT groups were 11.58 ± 0.57, 13.90 ± 0.17, and 15.36 ± 0.5, respectively. As demonstrated by the results, zirconia might benefit from the addition of more translucent porcelain to mimic the translucency of enamel.

In this study, ∆E values of the zirconia specimens on A2 and three other shades of abutments were obtained to evaluate shade-masking ability. The results showed significant differences in CIE L*a*b* and ΔE values, according to ceramic thickness and background type. Choi and Razzoog studied the masking ability of zirconia against four backgrounds and found a greater ΔE value with the black background than with the tooth-colored background [30]. This result is in accordance with that of our study. All evaluated ceramic materials showed acceptable masking ability against a simulated normal dentin-colored (ND3) substrate. However, no ceramic specimens, except the thicker HT specimens, could mask the severely discolored dentin background (ND9).

Several previous studies have found that not only abutment shade but also ceramic thickness affects the final shade of the restoration [12,16,25]. Tabatabaian et al. reported that greater thickness is needed to mask darker shades [31]. They concluded that for optimal masking, the minimum thickness of a zirconia coping should be 0.4 mm for A1- and A3.5-shade composite resins, 0.6 mm for amalgam, and 0.8 mm for nickel-chromium alloy abutments. This result is also consistent with that of our study. In the HT group, 1.5 mm thick specimens showed better masking ability than 0.8 mm thick ones on the ND9 background.

Concerning masking ability on the ND3 background, 0.8 mm thick specimens of the HT, ST, and UT groups showed optimal masking ability; however, the mean ΔE value of the specimens in the RS group (3.36) was above 2.6, i.e., it did not show ideal masking ability. The transparencies of the 0.8 mm thick specimens in the UT group were approximately 80% of those in the RS group; hence, UT could be an acceptable alternative to RS. In the ST and UT groups, a thickness of 1.5 mm was required to mask the titanium background, and ΔE values were similar to those of the RS group. The transparencies of 1.5 mm thick specimens in the UT group and similar specimens in the ST group were almost 90% and 80%, respectively, that of specimens in the RS group. Considering both transparency and masking ability, UT and ST could be good alternatives for restorations against titanium abutments. Overall, the results of this study showed that 0.8 mm thick UT specimens showed masking ability and TP almost similar to those of RS specimens of similar thickness. Choosing the correct ceramic material with appropriate thickness might be the best method for masking background color, and the decision should be made based on mechanical and esthetic requirements.

A limitation of this in vitro study was that it did not consider the effects of dental cements. Malkondu reported that regardless of cement type, color changes occur after cement application to monolithic zirconia [25]. Transparency and masking ability might vary depending on the color of the cement used between abutment and ceramic restoration. Further studies are needed to determine the effects of cement type and thickness on the final shade of ceramic restorations.

## 5. Conclusions

Within the limitations of this in vitro study, the following conclusions could be drawn:

1. A decrease in zirconia thickness from 1.5 to 0.8 mm significantly increased translucency; however, all specimens showed translucency lower than that of human enamel.
2. The TP value increased significantly with an increase in yttria content (mol %) of zirconia.
3. 5Y-ZP showed, approximately, 80% translucency at 0.8 mm thickness and 89% at 1.5 mm thickness, compared to that of lithium disilicate.
4. Monolithic CAD-CAM ceramics could mask a normal dentin background but could not mask severely discolored dentin at either 0.8 or 1.5 mm thicknesses.

## Figures and Tables

**Table 1:** Tested materials.

Material	Zirconia						Lithium Disilicate	
	3Y-TZP		4Y-ZP		5Y-ZP			
Product	Katana HT		Katana STML		Katana UTML		Rosetta SM	
Thickness (mm)	0.8	1.5	0.8	1.5	0.8	1.5	0.8	1.5
N	10	10	10	10	10	10	10	10

3Y-TZP, 3 mol % yttria-stabilized tetragonal zirconia polycrystal; 4Y-ZP, 4 mol % yttria-stabilized zirconia polycrystal; 5Y-ZP, 5 mol % yttria-stabilized zirconia polycrystal.

**Table 2:** Values of transparency parameters of specimens in the tested groups.

Group	0.8 mm Thickness			1.5 mm Thickness		
	Mean± SD	Median	Translucency Relative to the RS Group (%)	Mean± SD	Median	Translucency Relative to the RS Group (%)
HT	11.58 ± 0.57 ^a^	11.48	59.95	7.75 ± 0.57 ^a^	7.72	54.40
ST	13.90 ± 0.17 ^b^	13.90	72.58	11.68 ± 0.23 ^b^	11.63	81.96
UT	15.36 ± 0.50 ^c^	15.30	79.90	12.64 ± 0.19 ^c^	12.62	88.94
RS	19.18 ± 0.29 ^d^	19.15	100	14.20 ± 0.39 ^d^	14.19	100

SD, standard deviation. HT, Katana HT; ST, Katana STML; UT, Katana UTML; RS, Rosetta SM. Letters a, b, c, d mean statistically significant differences between groups (*p* < 0.05).

**Table 3:** Color difference values (ΔE) against ND3-shade composite resin simulating normal dentin.

Group	Mean ± Standard Deviation	
	0.8 mm Thickness	1.5 mm Thickness
Katana HT	1.77 ± 0.45 ^a^	0.91 ± 0.17 ^a^
Katana ST	2.16 ± 0.47 ^ab^	1.53 ± 0.35 ^b^
Katana UT	2.40 ± 0.27 ^b^	1.59 ± 0.19 ^b^
Rosetta SM	3.36 ± 0.24 ^c^	2.36 ± 0.14 ^c^

^a, b, c, d^ statistically significant differences between groups (*p* < 0.05).

**Table 4:** Color difference values (ΔE) against milled titanium simulating titanium abutment.

Group	Mean ± Standard Deviation	
	0.8 mm Thickness	1.5 mm Thickness
Katana HT	5.08 ± 0.26 ^a^	3.13 ± 0.32 ^a^
Katana ST	6.27 ± 0.62 ^b^	4.68 ± 0.39 ^b^
Katana UT	6.57 ± 0.52 ^bc^	4.91 ± 0.34 ^b^
Rosetta SM	6.59 ± 0.21 ^c^	4.71 ± 0.13 ^b^

^a, b, c, d^ statistically significant differences between groups (*p* < 0.05).

**Table 5:** Color difference values (ΔE) against ND9-shade composite resin simulating severely discolored dentin.

Group	Mean ± Standard Deviation	
	0.8 mm Thickness	1.5 mm Thickness
Katana HT	7.56 ± 0.33 ^a^	4.33 ± 0.45 ^a^
Katana ST	8.78 ± 0.61 ^b^	6.44 ± 0.31 ^b^
Katana UT	9.50 ± 0.55 ^c^	6.99 ± 0.35 ^c^
Rosetta SM	11.71 ± 0.28 ^d^	7.90 ± 0.31 ^d^

^a, b, c, d^ statistically significant differences between groups (*p* < 0.05).
